# *FOXP3* knockdown inhibits the proliferation and reduces NOTCH1 expression of T cell acute lymphoblastic leukemia cells

**DOI:** 10.1186/s13104-018-3700-3

**Published:** 2018-08-13

**Authors:** Satoru Yonekura, Mai Itoh, Erika Shiratori, Mika Ohtaka, Shuji Tohda

**Affiliations:** Department of Laboratory Medicine, Tokyo Medical and Dental University, Yushima 1-5-45, Bunkyo-Ku, Tokyo, 113-8519 Japan

**Keywords:** FOXP3, NOTCH1, T-lymphoblastic leukemia, Small interfering RNA

## Abstract

**Objective:**

Forkhead box P3 (FOXP3) is a master transcriptional factor of regulatory T-cells (Tregs). Recent studies have shown that FOXP3 is associated with growth inhibition of cancer cells. However, the role of FOXP3 in acute T-lymphoblastic leukemia (T-ALL) cells is not known. It was also reported that NOTCH signaling promoted the expression of FOXP3 in Tregs. However, the effect of FOXP3 on NOTCH expression in T-ALL cells is little known. Therefore, we examined the effect of *FOXP3* knockdown on the proliferation of T-ALL cells and NOTCH1 signaling.

**Results:**

Two T-ALL cell lines Jurkat and KOPT-K1, harboring activating *NOTCH1* mutations, were transfected with small interfering RNA against *FOXP3*. Cell growth was assessed with a colorimetric assay and morphology was observed under a microscope. *FOXP3* knockdown significantly reduced cell growth and induced morphological changes suggesting apoptosis. Quantitative polymerase chain reaction revealed that *FOXP3* knockdown caused the downregulation of mRNA expression of *NOTCH1* and *HES1*. These findings suggest that FOXP3 supports the growth of T-ALL cells although this can not be generalized because we examined only two cell lines. The observed growth suppression can be partly due to the downregulation of NOTCH1 signaling. FOXP3 may be a potential therapeutic target in T-ALL.

## Introduction

T cell acute lymphoblastic leukemia (T-ALL) is a highly aggressive disease caused by malignant transformation of early T cell progenitors [1], characterized by mutation of *NOTCH1* [1, 2]. NOTCH1 signaling pathway is essential for self-renewal and differentiation in hematopoietic stem cells [3]. Consequently, NOTCH1 direct target genes and NOTCH1-regulated gene expression have been rigorously studied for elucidating the oncogenic mechanism in T-ALL [1].

Forkhead box P3 (FOXP3) is a master transcriptional factor for the development of regulatory T-cells (Tregs) [4]. In addition to Tregs, the expression of FOXP3 is reported in various cancer cell lines including Jurkat, a T-ALL cell line [5]. Upregulation of FOXP3 is associated with inhibition of cell growth in solid tumor cell lines from breast [6], prostate [7], epithelial ovarian [8] and gastric [9] cancer. However, the role of FOXP3 on the proliferation of T-ALL cells is not clear yet. To assess the effect of FOXP3 knockdown on cell growth, we introduced small interfering RNA (siRNA) against *FOXP3* in T-ALL cell lines.

Several studies show the upregulation of FOXP3 by NOTCH signaling in Tregs: indeed, NOTCH ligands induce the differentiation of CD4+ CD25+ Tregs [10, 11] and NOTCH signaling promotes the expression of FOXP3 in Tregs [12, 13]. However, little is known about the relation between FOXP3 and NOTCH1 in T-ALL cells. A recent study has shown that a γ-secretase inhibitor that blocks NOTCH activation reduces the expression of FOXP3 in Jurkat cells [14]. We decided to examine whether, on the other hand, FOXP3 has an effect on NOTCH1 in T-ALL cells. Therefore, we investigated the expression of *NOTCH1* and hairy and enhancer of split-1 (*HES1*), a *NOTCH1*-targeted gene, after knockdown of *FOXP3*.

## Main text

### Methods

We used two T-lymphoblastic leukemia cell lines, Jurkat and KOPT-K1, harboring activating *NOTCH1* mutations [2]. Jurkat cells were purchased from the European Collection of Cell Cultures (Porton Down, Wiltshire, UK), and KOPT-K1 cells were donated by Drs. Harashima and Orita (Fujisaki Cell Center, Okayama, Japan). FOXP3 knockdown was performed by transfecting small interfering RNA (siRNA) targeting *FOXP3* into the cells. Three different pre-designed siRNAs (Stealth siRNA™) targeting *FOXP3* (HSS 121456, 121458, and 181786) were purchased from Life Technologies (Carlsbad, CA, USA). Stealth RNAi negative control Duplex (Life Technologies) was used as a control. Cells were transfected with 80 nM of each siRNA using the Neon™ pipette tip chamber-based electroporation system (Life Technologies) according to the manufacturer’s instructions, and immediately transferred to culture medium.

To assess cell proliferation, we used the colorimetric WST-8 assay (Dojindo Laboratories, Kumamoto, Japan). Cells transfected with *FOXP3* siRNA or control siRNA were cultured in RPMI-1640 supplemented with 10% fetal bovine serum in 96-well culture plates in a humidified 5% CO_2_ atmosphere for 5 days. Then, WST-8 and 1-methoxy-5-methylphenazinium methyl sulfate were added, and optical density (OD) was measured with an ELISA plate reader. Relative cell proliferation was calculated as the percentage of the mean OD value normalized to that of the control. The effects of the siRNA on cell morphology and apoptosis were examined in cytospin preparations stained with Wright’s stain and observed under a microscope.

To evaluate the effect of *FOXP3* knockdown on mRNA expression, RNA was extracted from the cells 4 h after the electroporation using the High Pure RNA isolation kit (Roche Diagnostics, Mannheim, Germany) and used for first-strand cDNA synthesis. The effects of *FOXP3* silencing on gene expression were examined by quantitative polymerase chain reaction (qPCR) in a LightCycler (Roche Diagnostics) using a FastStart DNA Master SYBR Green I kit, LightCycler primer sets (Roche Diagnostics) and QuantiTect Primer Assays (Qiagen, Hilden, Germany) according to the manufacturer’s instructions. The expression of each mRNA was normalized to that of β-actin (*ACTB*) mRNA, which we measured concurrently.

All statistical analyses were performed using the R freeware (http://www.r-project.org). For intergroup comparisons, we used the Student’s t-test. Correlation analysis was performed using Spearman’s rank correlation coefficient (rho). All P-values were two-sided, and P-values ≤ 0.05 were considered statistically significant. We used the GraphPad Prism 7.00 software (GraphPad Software Inc., La Jolla, CA, USA) to generate all graphs. Data was presented as the mean ± standard error of the mean.

### Results and discussion

**Fig. 1:**
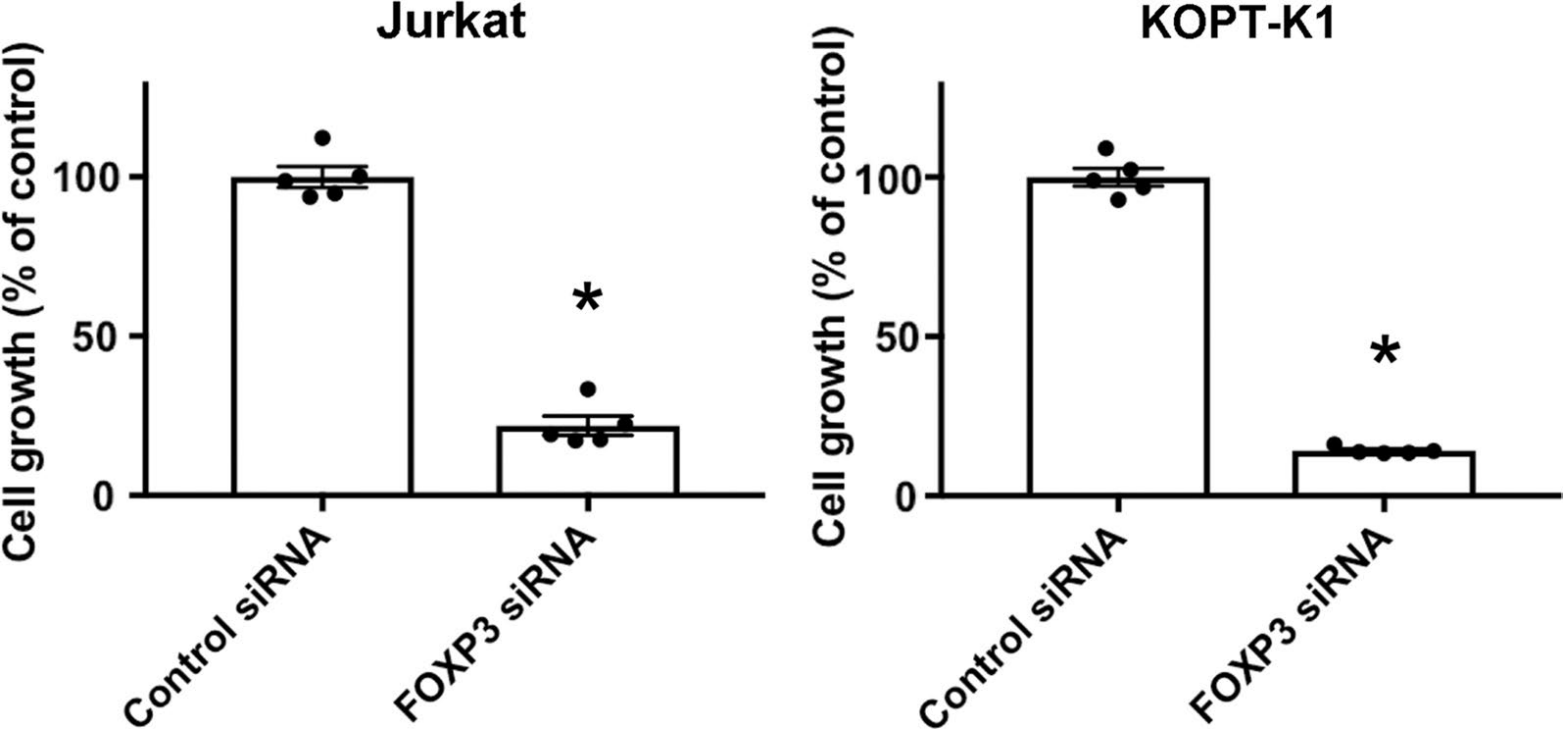
Effect of *FOXP3* knockdown on the growth of T-cell acute lymphoblastic leukemia cell lines. Five days after *FOXP3* silencing, cell growth was assessed using a colorimetric assay. Cell growth is shown as a percentage of the mean OD value in *FOXP3*-silenced normalized to that of control cells ± standard error (n = 5). *P < 0.05 compared to control cells

**Fig. 2:**
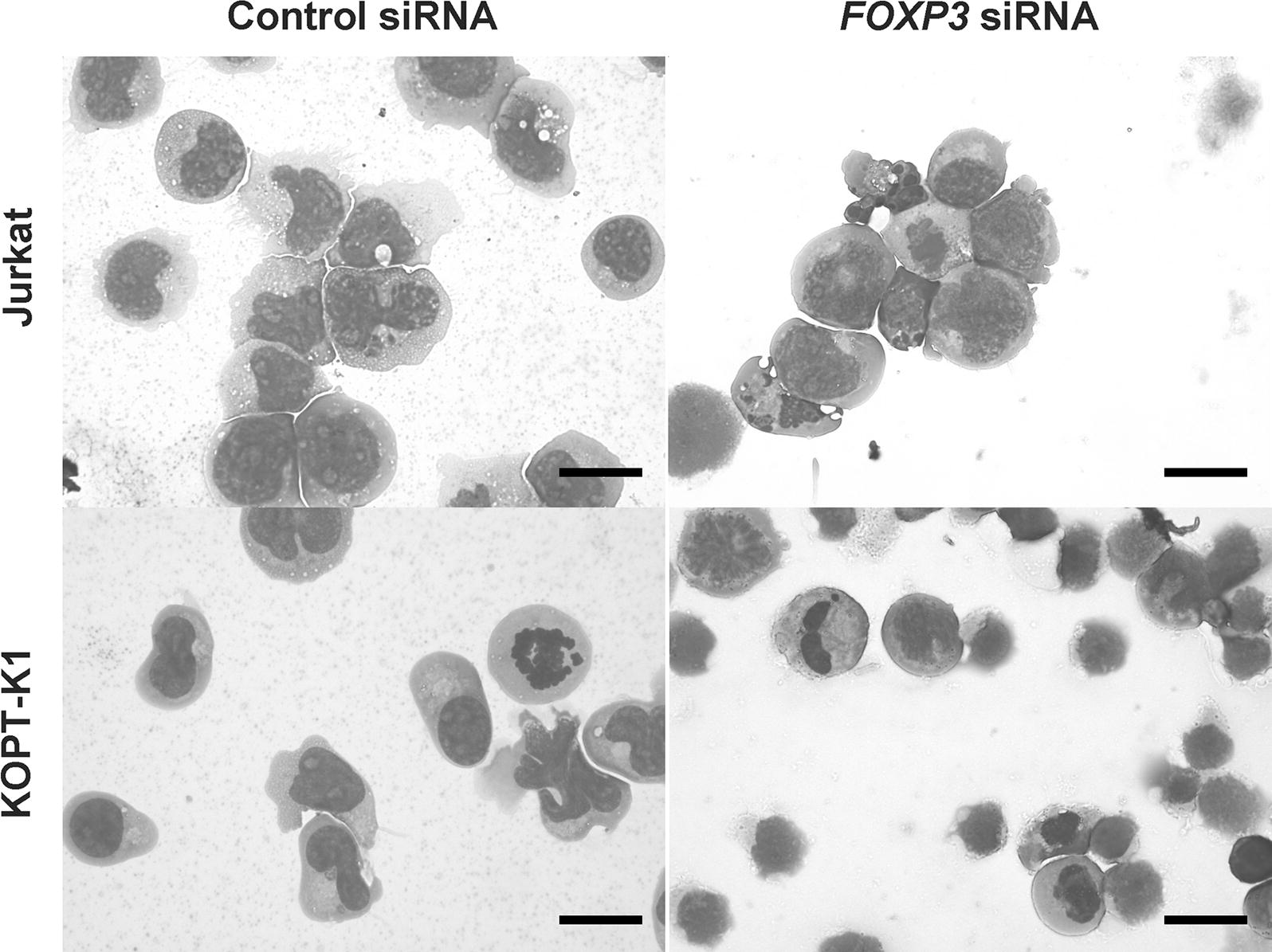
*FOXP3* knockdown induces apoptosis in Jurkat cells and KOPT-K1 cells. Cytospin preparations from *FOXP3*-silenced or control cells were cultured for 6 h and stained with Wright’s stain (original magnifications, ×600). Scale bar: 20 µm

We knocked down *FOXP3* expression by introducing siRNA against *FOXP3* in Jurkat and KOPT-K1 cells. The most potent siRNA against *FOXP3* was HSS121458 (5′-CCGGAUGUGAGAAGGUCUUCGAAGA-3′). Upon RNA interference, the expression of *FOXP3* was significantly reduced to 28.6% ± 3.2% and 44.5% ± 18.7% in Jurkat and KOPT-K1 cells, respectively, as shown by qPCR (Table 1). Expression of *FOXP3* had a trend to correlate with expression of *NOTCH1* in Jurkat (rho = 0.771, P = 0.103) and KOPT-K1 (rho = 0.829, P = 0.06). To evaluate the effect of FOXP3 on the cell growth, we performed proliferation assays 5 days after RNA interference (Fig. 1). Cell growth was significantly suppressed to 21.9% ± 3.01% and 14.2% ± 0.51% in Jurkat and KOPT-K1 cells, respectively. Additionally, observation of cytospin preparations indicated that apoptotic cells with nuclear condensation and apoptotic bodies appeared upon silencing of *FOXP3* in Jurkat cells and KOPT-K1 cells (Fig. 2).

**Table 1:** *FOXP3*, *NOTCH1* and *HES1* mRNA expression in *FOXP3* silenced T-ALL cells

	Jurkat	KOPT-K1
*FOXP3*	28.6 ± 3.2%*	44.5 ± 18.7%*
*NOTCH1*	55.5 ± 3.7%*	59.9 ± 9.3%*
*HES1*	52.0 ± 18.7%*	34.0 ± 11.3%*

RNA was extracted from Jurkat or KOPT-K1 cells transfected with control siRNA or *FOXP3* siRNA (HSS121458) after 4 h. The expression of *FOXP3, NOTCH1* and *HES1* was measured by qPCR and normalized to that of *ACTB*. The values indicate the expression in *FOXP3* silenced cells relative to that in control cells as the mean ± standard error of the mean (n = 3)

* P < 0.05 compared to the gene expression in control cells

Because knockdown of *FOXP3* inhibited cell growth and induced apoptosis, we hypothesized that FOXP3 is associated with the expression of genes that induce proliferation of T-ALL cell lines. To understand if this was the case, we investigated the expression of *NOTCH1* and *HES1* in *FOXP3*-silenced cells. We found that the expression of these genes was significantly reduced to almost 50% in Jurkat and KOPT-K1 cells compared with control cells (Table 1).

The current study showed that silencing of *FOXP3* caused growth suppression and apoptosis. The upregulation of *FOXP3* has been reported to inhibit cell growth in several solid tumor cell lines [6–9]. However, we found opposite results. *FOXP3* promotes tumor proliferation in melanoma [15], thyroid cancer cells [16] and radiation-induced T-cell leukemia in Balb/c mice [17]. The published data are inconsistent even in the same tumor cell lines such as breast and gastric cancer [18]. We believe that the role of FOXP3 on cell growth can diverge in each cell lines due to different mechanism of oncogenesis or proliferation. We found that knockdown of *FOXP3* reduced *NOTCH1* expression in T-ALL cell lines. The observed growth suppression and apoptosis can be partly due to the downregulation of NOTCH1 signaling because NOTCH1 plays a major role in the growth of T-ALL cells. Previous studies have shown that NOTCH1 induces the expression of FOXP3 in Tregs. Samon et al. showed that NOTCH1 and transforming growth factor (TGF)-β1 cooperatively support the expression of FOXP3 and the maintenance of peripheral Tregs by allowing the binding of NOTCH signaling-related molecules to the promoter of *FOXP3* [13]. In addition, Luo et al. reported that inhibition of NOTCH1 reduced FOXP3 expression in Jurkat cells [14]. Conversely, our study showed that FOXP3 affected NOTCH1 expression in T-ALL cell lines.

Our study has clinical relevance because it points to FOXP3 as a new therapeutic target in T-ALL. Suppression of FOXP3 in T-ALL can lead to both reduced cell growth and apoptosis. In addition, expression of FOXP3 in cancer cells might be associated with immunosuppression. For instance, Karube et al. reported that FOXP3-positive adults with ATLL suffered from severe infectious diseases more often than the FOXP3-negative patients [19]. On the same note, FOXP3 expression in cancer cells might represent a tumor escape mechanism [20, 21]. In hematopoietic malignancy, lymphoma cells expressing FOXP3 in ATLL patients evaded host immune response including cytotoxic T lymphocytes [22]. Therefore, the targeting of FOXP3 in T-ALL might not only affect the proliferation of tumor cells but also improve the immunosuppressive status of the patients with T-ALL.

In summary, *FOXP3* knockdown inhibits the growth and reduced NOTCH1 expression of T-ALL cells. Reduced NOTCH1 signaling can be one of the mechanisms of the growth suppression. Therefore, FOXP3 may be a potential therapeutic target in T-ALL.

## Limitations

We suggested that FOXP3 support the growth of T-ALL cells. However, this can not be generalized in T-ALL cells because we just examined only two T-ALL cell lines. To show the universality of this phenomenon, we have to examine other T-ALL cell lines and T-ALL cells from patients in the future.

We also showed that *FOXP3* knockdown induced apoptosis based on morphological changes of the cells. We tried to show apoptosis at the protein level with immunoblot analysis for caspase-3 activation or flow cytometry for phosphatidylserine externalization. However, we could not obtain the clear results because siRNA induction rapidly caused cell death and degradation of proteins including α-tubulin. We also have to show apoptosis of the cells with some other methods in the future.

## Abbreviations

FOXP3: Forkhead box P3
Tregs: regulatory T-cells
T-ALL: acute T-lymphoblastic leukemia
siRNA: small interfering RNA
